# The Application of the Philosophical Thinking of “Three” From I Ching to Medical Education

**DOI:** 10.3389/fmed.2021.759282

**Published:** 2021-12-16

**Authors:** Yingjie Du, Xinqing Zhang, Jinjing Zhang, Guyan Wang

**Affiliations:** ^1^Department of Anesthesiology, Beijing Tongren Hospital, Capital Medical University, Beijing, China; ^2^School of Humanities and Social Sciences, Chinese Academy of Medical Sciences and Peking Union Medical College, Beijing, China; ^3^Department of Anesthesiology, First Affiliated Hospital of Jinzhou Medical University, Jinzhou, China

**Keywords:** “Three”, I Ching, dialectical thinking, One Dividing into Three, medical education

## Abstract

Since ancient times, the Chinese have had a special understanding of the “Three”. Chinese philosophy originates from the I Ching, and the philosophical concept of “Three” is the core of the I Ching. The philosophical thinking about “Three” entails a complete dialectical thinking method that is consistent with the Western philosophical concept of “One Dividing into Three”. In this paper, we explain the philosophical concept of “Three” and suggest its application to medical education, including the learning and application of new technology, shared decision making between doctors and patients, and integration of medical humanities and medical science.

## Introduction

“Three” is the number most used by the Chinese, but few people pay attention to it; they use it every day without thinking in depth about what it means. There are many idioms using “Three” in Chinese, and “Three” is often an abstract concept rather than a real one. It does not represent the original meaning of the number itself but has different meanings in different contexts (1). In some situations, “Three” can represent many times or most, such as “A man has three heads and six arms”, “One shouldn't make the same mistake more than three times”, and “One day apart seems like three autumns”. In other situations, “Three” can express a small amount, such as “make one thing clear with three words or two words” and “one cannot leave one's own profession in three sentences”. Since ancient times, the Chinese have been used to autonomously enter the realm of ‘Three”. As such, the philosophical concept of “Three” is deeply rooted in the marrow and blood of every Chinese person.

What philosophical thinking is contained in “Three” from the I Ching? What is the specific importance of philosophical thinking of “Three” for medical education? Despite its long-standing contribution to studies on education in other professions, philosophy has been absent from the discussion of medical education (2). In this paper, we explained the philosophical concept of “Three”, and we suggested the application of the philosophical concept of “Three” from I Ching to medicine education, including the learning and application of new technology, shared decision making between doctors and patients, and integration of medical humanities and medical science.

## Discussions

### The Philosophical Concept of “Three” Is Complete Dialectical Thinking

#### “Three” Is the Core Content of the I Ching

All wisdom of the Chinese is developed from nature; that is, the Chinese learn from nature and take nature as their teacher. In Chinese myths and legends, ancient Fuxi mainly used three natural methods of looking up, looking down and looking around nature to penetrate the code of the heaven, and humans were united as one, which is Yin and Yang. Yin and Yang are the most basic elements that constitute everything in the universe. They can be distinguished as positive and negative elements, but they cannot be separated. This state of Yin and Yang is “Tao”, and the so-called “one Yin and one Yang is Tao”. In the middle of Yin and Yang, there is an independent third part that connects the opposing sides. Some scholars call this the “Interaction of Yin and Yang” (3). In this article, we call it “Three”. “Three” is the foundation of natural development. Since ancient times, the Chinese people have known that only with the interaction of Yin and Yang will there be sustainable development of nature. The philosophical concept of “Three” established the theory of Tai Chi and the thinking of I Ching. Only by viewing Yin and Yang as three parts can we understand the profound connotation of I Ching. Therefore, the Chinese people have a special feeling about “Three” and a unique understanding of the meaning of “Three”, and they regard “Three” as the root of the development and change of everything.

The ancient Chinese believed that “Three” was perfect, and there was a kind of natural worship for “Three”. In ancient times, Fu Xi painted many hexagrams, but he ultimately decided to hand down the three hexagrams. The Three-Character Classic notes that “Three” is the mathematical basis of heaven, earth, and humans. In the late Spring and Autumn period, Laozi proposed the metaphor of “Three”: “Tao gives birth to one, one gives birth to two, two gives birth to three, and Tao gives birth to all things”. “Three” is endowed with the greatest generality and abstraction (4).

#### “One Dividing Into Three” Is the Basic Frame Work of Western Philosophy

The Chinese are not the only culture obsessed with “Three”; “Three” is also of great significance in Western philosophy. Pythagoras, the Greek philosopher and mathematician who was the first Western pioneer in the systematic study of “Three”, believed that everything was determined by the ternary. The ancient Greek philosopher Aristotle noted that the entity had the third degree, and anything with a form had no volume without “Three”, i.e., any entity is determined by three-dimensional space. The ancient Greek philosopher Prokhora first used the development of the three-part law and three paragraphs to establish the trinity of the philosophical system of idealism, which had a great impact on later Hegelian philosophy. In Hegel's philosophy, “One Dividing into Three” has the most speculative nature and the most universal meaning. “Three” also represents three-dimensional space. The world is thought to consist of the earth, the sea and the sky. Nature is made up of animals, plants and minerals. Western philosophy holds that the human body has the triple nature of body, mind and spirit. Coincidentally, Kepler's three laws of planetary motion, Newton's three laws of the mechanical motion of objects, and the three golden laws of the twentieth century in the West all contain “Three”. To Westerners, “Three” has both rationality and divinity and is the embodiment of nobility, auspiciousness and perfection.

#### Philosophical Concept of “Three” and Philosophy of “One Dividing Into Three”

The philosophical concept of the “Three” has an objective historical basis and is closely related to the realistic three-dimensional world. The ternary structure conforms to the mechanism of human understanding of nature, which can be easily perceived and accepted by people. It can form various intermediate components that have both poles and contain the transformation of the two, drawing attention to the long gray zone between them (5). In both the East and the West, although there are many ways of thinking, people are accustomed and willing to use the philosophical concept of “Three” in our work and life. The law of development of things expressed by the changes of Yin and Yang from the I Ching is a complete dialectical thinking method. The Chinese philosophical concept of “Three” is a kind of wisdom about choice (6). “Three” is the first complete unit of the occurrence of things, the base of the generation and development of all things, and the most basic unit in all natural fields, including medicine (7).

Simple dichotomy cannot satisfy people regarding the understanding of the material world because while two sides may be opposed to each other as extreme opposites, there is a gray area between them. Dichotomy is clearly insufficient to describe the transition state, and the intermediate stage is the philosophical concept of “Three” (8). The philosophical concept of “Three” follows the law of the unity of opposites, and relative changes are used to understand the unity of things such as Yin, Yang and the “Three” from the I Ching. The philosophical concept of “Three” regards “Two” as “Three” to turn a two-step problem into a three-step solution and find a suitable point to balance opposing sides. Therefore, “Three” is a kind of balance, also known as “The Doctrine of the Mean” in traditional Chinese culture. Because truth does not exist in either extreme but in the middle of them, we call this “Three”. “Three” represents thousands of years of Chinese cultural accumulation, the formation of both poles and the formation of an infinite number of intermediate elements so that the philosophical concept of the “Three” can be handed down to the present (9).

The philosophical concept of “Three” from I Ching is essentially consistent with the concept of “One Dividing into Three” in Western philosophy. Some foreign scholars also believe that balance is central to this worldview: all objects or processes are considered to be composed of two complementary principles. Yin and Yang are interdependent opposites such that it is not possible to have light without dark or good without evil (10). Of course, the emphasis on the philosophical concept of “Three” is the fundamental method of dialectics. This does not mean that the dialectical method only involves “One Dividing into Three”; in different circumstances, it may be “One Dividing into Four”, “One Dividing into Five”, etc. We argue that the philosophical concept of “Three” prompts us to improve the level of our thinking. Currently, the philosophical concept of “Three” stimulates people's cognitive potential to satisfy the requirements of medical development in the new century.

### Application of the Philosophical Concept of “Three” to Medical Education

#### Application of the Philosophical Thinking of “Three” to the Learning and Application of New Technologies

The development and progress of medical technology have brought great benefits to human health. In the face of numerous emerging new technologies, effectively transforming them into clinical applications requires the support of the philosophical concept of “Three”. We should advocate science but not blindly believe in it; it is necessary to continue to develop science in the critical use of new technology and promote the positive development of modern medicine. However, some doctors tend to simply accept whatever is scientifically proven to be correct, accept new technologies without criticism, blindly pursue new technologies while ignoring their actual clinical application value, and only pursue the benefits while ignoring the negative results that may be caused by these technologies. These phenomena reflect a narrow two-dimensional way of thinking. The philosophical concept of “Three” is not to blindly follow the current technology but to use philosophical thinking and critical vision to examine things for higher development. Therefore, the establishment of the philosophical concept of “Three” represents a new trend in medical education in the 21st century.

Good clinicians should adhere to evidence-based medicine in the face of new technologies, and this process reflects the philosophy of “Three”. When Professor David Sackett, a noted clinical epidemiologist, first published “Evidence Based Medicine: How to Practice and Teach Evidence Based Medicine” in 1997, he defined evidence-based medicine as the careful, accurate and judicious use of the best available research evidence to determine a patient's treatment (11). However, in the revised version in 2000, Sackett proposed a new definition: carefully, accurately and judiciously use the best available evidence, combine the professional skills and clinical experience of doctors, and consider the values and wishes of patients to develop a specific treatment plan based on the organic combination of these three factors (12–14).

In clinical practice, when learning and applying new technologies, we should apply the philosophical thinking of “Three” in them. Even if there are literature reports that the clinical effect of a certain technology is good, it may not actually be good. It may be effective only for a specific patient in combination with the patient's own experience. For the application of new technology in clinical practice, we should not blindly pursue novelty and should avoid indulging in narrow personal experiences. Instead, we should use the philosophical thinking of “Three” and organically combine the experimental evidence, doctor's personal experience and patient's response.

#### Application of the Philosophical Concept of “Three” to Shared Decision Making Between Doctors and Patients

Currently, in China, a dispute remains over the doctor–patient cooperative medical model. The focus of this dispute is whether the decision-making power for medical treatment is dominated by the patient or the doctor (15). Compared with Western countries, China has a gap in this regard and can learn from the West.

The traditional Chinese concept is a physician-led diagnosis and treatment mode because doctors know medical knowledge and how to treat diseases. However, because the trust between doctors and patients has been seriously reduced, the medical model that solely centers on doctors has been severely challenged. Therefore, some doctors advocate a patient-centered approach, in which patients make all medical decisions to eliminate the dilemma of doctor–patient conflict. This advocacy obviously goes from one extreme to the other, and irresponsible behavior in either doctor-centered or patient-centered mode is a kind of dichotomous thinking. Although it has significance in a temporary social environment, it cannot adapt to the current medical mode. In a complex social medical treatment environment, the key is not to determine who is in charge of the problem but to build a harmonious connection between doctor and patient. In both Chinese and Western clinical medicine, a mutual participation model of “shared decision-making between doctors and patients” is becoming the mainstream direction. Shared decision making is defined as “a formal process or tool that helps physicians and patients work together to choose the treatment option that best reflects both medical evidence and the individual patient's priorities and goals for his or her care”. Patients express their expectations for disease treatment and risk acceptance to doctors, and doctors and patients exchange opinions and reach a consensus on problems in diagnosis and treatment. This model is characterized by doctors and patients having equal rights and status; the two sides cooperate with each other in medical decision-making and its implementation (16). The purpose of shared decision making is to share responsibilities between doctors and patients, improve the compliance and positive attitudes of patients toward cooperation with treatment, and promote the formation of more reasonable clinical decision-making.

The process of “shared decision making” embodies the philosophical concept of “Three”. It is not a purely technical issue but involves combining technology and humanities, and it is a social practice of cooperative doctor–patient decisions. When we practice “shared decision making”, we should apply the philosophical concept of “Three” and implement the organic integration of two as one, i.e., the combination of doctors who have mastered medical knowledge and patients who require treatment. Doctors and patients should reach a consensus to achieve the purpose of scientific diagnosis and treatment to enhance the understanding and trust between doctors and patients, eliminate the estrangement between doctors and patients, and establish a harmonious doctor–patient relationship.

#### Application of the Philosophical Concept of “Three” to Integrate Medical Humanities and Medical Science

The progress of science and technology has promoted the process of medical development. Contemporary medicine involves a balance between technology and humanity, which represents an unprecedented period of directional choice for medical development. Medical technology brings hope to people but may also produce panic and worry, and the tension and balance between medical science and humanism are increasingly lost. The rationality of medicine is completely under the control of the development needs of technology, the medical characteristics of benevolent subjectivation are quietly resolved by technology, and medicine is lost on the path of the blind pursuit of technology itself. Simultaneously, the absolutism of technology pushes single biomedical science to the extreme, further exposes its defects and deficiencies and gradually moves medicine in an increasingly deformed and one-sided direction. However, when technology-themed negative behaviors are attacked, many doctors do not understand or even feel disgusted. They emphasize that there is only humanistic care without technology, so how can they treat patients? Although the ultimate development of technology can solve human problems, technology is the best humanized medical treatment. This is an irresponsible complaint and a typical error in thinking. One of the integrative themes of future medical education is a humanistic approach to patient safety that requires encouraging humanistic doctors (17). When we emphasize medical humanities, we do not underestimate the role of technology but make technology more effective. Estrangement between science and humanities is the most significant problem of our time.

To understand the relationship between technology and humanity using the philosophical concept of “Three”, simply going to either side is obviously a kind of extreme dichotomous thinking. Technology is the foundation of medical science, but doctors only have technology, and a lack of medical humanities is not sufficient. Science and technology alone cannot make life more meaningful; simultaneously, medicine with only humanity and a lack of technology is a step backward. Medical technology and medical humanities are not in opposition; there must be a third dependent relationship between them, i.e., the combination of technology and humanities, and it is necessary to maintain a certain tension relationship: the intersection of the “Three”.

Although great changes have occurred in medical humanistic education in China, improvements are necessary, and Chinese medical educators must learn more from Western practices (18). In medical education, we should promote the integration of the scientific spirit and humanistic spirit of medicine (19, 20). The meaning of the humanities for technology is to correct the directional bias in technology and resist the irrational expansion of a lack of morality (21). Through concrete practice and conveying love and care for life, humanity cannot be separated from technology, and technology must rely on humanities. Some scholars suggest the integration of arts and humanities in medical education (22).

Therefore, the key for medicine to escape the predicament of modernity lies in the rational integration of the tension between technology and humanity. Medicine requires technology, but it should maintain better control of technology. It cannot make technology the main body of medicine. The balance of tension between medical humanities and medical science is the philosophy of “Three”.

## Conclusions

Since ancient times, the Chinese have had special feelings about the “Three”. Chinese philosophy originates from the I Ching, “Three” is not only a simple number but has also been endowed with profound cultural connotations. The philosophical concept of “Three” is a complete dialectical thinking method that is consistent with the Western philosophical concept of “One Dividing into Three”. In the era of the biological-psychological-social medicine model, we can apply the philosophical concept of the “Three” to the learning and application of new technology, the process of shared decision making between doctors and patients and the integration of medical humanities and medical science.

## Author Contributions

YD and XZ wrote the preliminary manuscript. JZ and GW reviewed and revised the manuscript. All authors approved the final version.

## Funding

This study was supported by the Beijing Hospitals Authority Clinical Medicine Development of Special Funding Support (No. ZYLX202103).

## Conflict of Interest

The authors declare that the research was conducted in the absence of any commercial or financial relationships that could be construed as a potential conflict of interest.

## Publisher's Note

All claims expressed in this article are solely those of the authors and do not necessarily represent those of their affiliated organizations, or those of the publisher, the editors and the reviewers. Any product that may be evaluated in this article, or claim that may be made by its manufacturer, is not guaranteed or endorsed by the publisher.
